# Retraction: The pomace extract Taurisolo protects rat brain from ischemia-reperfusion injury

**DOI:** 10.3389/fncel.2026.2026335

**Published:** 2026-10-26

**Authors:** Frontiers Editorial Office

The Journal retracts the 28 January 2020 article cited above.

Following publication, concerns were raised regarding the integrity of the images in the published figures. The authors failed to provide a satisfactory explanation during the investigation, which was conducted in accordance with Frontiers' policies. As a result, the data and conclusions of the article have been deemed unreliable and the article has been retracted.

The retraction was approved by the Chief Editors of Frontiers in Cellular Neuroscience and the Chief Executive Editor at Frontiers. The authors were informed of the retraction and had an opportunity to respond. This communication has been recorded by the publisher.

